# Single cell sequencing analysis constructed the N7-methylguanosine (m7G)-related prognostic signature in uveal melanoma

**DOI:** 10.18632/aging.204592

**Published:** 2023-03-14

**Authors:** Jiaheng Xie, Liang Chen, Yuan Cao, Chenfeng Ma, Wenhu Zhao, JinJing Li, Wen Yao, Yiming Hu, Ming Wang, Jingping Shi

**Affiliations:** 1 Department of Burn and Plastic Surgery, The First Affiliated Hospital of Nanjing Medical University, Nanjing 210029, Jiangsu, China; 2 Department of Hepatobiliary Surgery, Jiaxing First Hospital, Jiaxing 314001, Zhejiang, China; 3 Fourth School of Clinical Medicine, Nanjing Medical University, Nanjing 210029, Jiangsu, China; 4 Department of Neurosurgery, The First Affiliated Hospital of Nanjing Medical University, Jiangsu Province Hospital, Nanjing 210029, Jiangsu, China; 5 Hepatobiliary/Liver Transplantation Center, First Affiliated Hospital, Nanjing Medical University, Nanjing 210029, Jiangsu, China; 6 Department of Ophthalmology, The First Affiliated Hospital of Nanjing Medical University, Jiangsu Province Hospital, Nanjing 210029, Jiangsu, China; 7 College of Pharmacy, Jiangsu Ocean University, Lianyungang 222005, Jiangsu, China

**Keywords:** uveal melanoma, N7-Methylguanosine, single cell sequencing analysis, PAG1, immune microenvironment

## Abstract

Background: Uveal melanoma is a highly malignant tumor in the eye. Its recurrence and metastasis are common, and the prognosis is poor.

Methods: The transcriptome data of UVM were downloaded from TCGA database, and the single cell sequencing dataset GSE139829 was downloaded from GEO database. Weighted co-expression network analysis was used to explore the modules associated with m7G. Lasso regression was used to construct M^7^G-related prognostic signature. Immune infiltration analysis was used to explore the significance of the model in the tumor immune microenvironment. Finally, cell assays were used to explore the function of key genes in the MUM-2B and OCM-1 cell lines of UVM.

Results: The prognostic signature was constructed by Cox regression and Lasso regression. Patients could be divided into high-risk group and low-risk group by this signature, and the high-risk group had worse prognosis (P<0.05). Cell experiments showed that the proliferation, invasion and migration ability of UVM cell lines were significantly decreased after the knockdown of *PAG1*, a key gene in signature, which proved that *PAG1* might be a potential target of UVM.

Conclusions: Our study explored the significance of m^7^G in UVM, provided biomarkers for its diagnosis and treatment.

## INTRODUCTION

Uveal melanoma (UVM) is the most common intraocular malignancy in adults [1]. This malignant tumor typically originates from the melanoma cells in the choroid, ciliary body, and iris, causing progressive loss of vision and visual distortion [2–4]. Typical signs of the disease are blurred and distorted vision and photopsia [5]. However, approximately 30% of the patients are asymptomatic and are diagnosed according to routinary ophthalmological tests [6]. Uveal melanoma is highly malignant and can be life-threatening once the tumor gets into an advanced stage, especially when hematogenous metastasis is involved [7]. Unfortunately, due to the latency of the symptoms, a large number of patients are unable to have access to proper treatment, especially in low and middle-income countries [8]. Treatments for Uveal melanoma mainly include surgical resection of the lesions, radiotherapy, and phototherapy [9]. In advanced cases, however, surgical enucleation is strongly recommended [10]. With the development of basic and clinical research, scientists are now increasingly aware of the underlying molecular mechanisms that give rise to UVM [11]. Targeted therapies and immunotherapies that mainly focus on the advanced stages of UVM are on the way [12, 13].

N7-Methylguanosine (m^7^G) is a commonplace post-transcriptional modification of mRNA, tRNA, rRNA, and lncRNA in eukaryotes, which plays an indispensable role in cell metabolism and vitality [14]. It should be noted that the m^7^G RNA modification in mRNA was only recently noted by scientists according to basic research [15]. Recently, m^7^G modification was found in tRNA in various types of malignancies, for example, intrahepatic cholangiocarcinoma, which was associated with an adverse outcome in patient survival [16]. Hence, finding the potentially clinically relevant m^7^G genes and the related mechanisms are becoming increasingly important in basic research in the realms of Uveal melanoma.

Now, rapid advances in bioinformatics have made it possible to further explore the prognostic value and immunological relevance of m7G in UVM [17]. It can provide reliable basis for diagnosis, prediction and treatment of UVM by exploring the data of genomics, proteomics, metabolomics and other aspects to further study its pathogenesis, evolution pattern and treatment trend [18–20]. These include: 1) Personalized cancer diagnosis, prediction, and treatment strategies based on genomic and proteomics research; 2) Using meta-spectral data combined with machine learning technology to establish more accurate prediction models and provide novel treatment plans; 3) Using systems biology principles to analyze network interactions in cancer; 4) Design more accurate diagnostic tools and new drugs based on multi-dimensional data such as cellular and molecular data. At present, many prognostic models of UVM have been constructed. For example, Luo et al. constructed a ferroptosis related prognostic model in UVM through transcriptome analysis to predict the prognosis of UVM patients, and found that high-risk patients had significantly worse prognosis, which may be related to Mast cells [21]. Chuah et al. built an autophagy related prognostic model in UVM through the analysis of GEO data set, and found that autophagy score was related to immune-related functional pathways and immune cell infiltration [22]. However, no M7G-related prognostic model has been constructed and validated in UVM. In addition, there are few studies related to single cell sequencing analysis of UVM. So, we did this study.

Here, we combined single-cell analysis, weighted Gene Co-expression network analysis (WGCNA), as well as a lasso-cox regression method to establish an m^7^G gene signature to predict survival and clinical outcomes in UVM patients and to identify the gene phosphoprotein membrane anchor with glycosphingolipid microdomains 1 (*PAG1*) that was most strongly associated with patient outcomes. Finally, *in vitro* experiments were used to validate our findings.

## MATERIALS AND METHODS

### TCGA data downloading and processing

UVM RNA-seq data were downloaded from the TCGA (https://portal.gdc.cancer.gov/) database. Data inclusion criteria were: (1) patients with prior pathological diagnosis of Uveal melanoma and (2) patients with detailed clinical information, including survival time, clinical stages, survival status, and so on. All data were processed and integrated using Perl software.

### WCGNA analysis for phenotype-associated modules and genes identification

Weighted gene co-expression network analysis (WCGNA) is an integrative method used for identifying modules and genes that are most significantly related to the sample phenotypes. A WGCNA analysis was performed using the WGCNA R package (version 3.2.2) to assess the relationship between clusters of co-expressed genes and the tumor-related phenotypes.

### Establishment of an m^7^G-related gene signature for prognosis

The method for establishing a prognostic signature has been intensively described in our previous articles. The least absolute shrinkage and selection operator (LASSO) regression was performed to construct a prognostic signature. The risk score can be illustrated as follows:


$$\begin{array}{c} \text{Risk}\;\text{score}=\sum_{i=1}^{n}\beta_{i}*gene\text{\_}expression\\ (\beta_{i}\;:coefficient\;of\;gene\;i;\;\\ gene\text{\_}expression_{i}\;:expression\;of\;gene\;i) \end{array}$$

The patients were grouped by risk scores, which divided them into high-risk and low-risk groups. Survival analysis was performed accordingly using package *Survival* (version 3.2).

### Single-cell sequencing and data processing

Single-cell sequencing data of Uveal melanoma *GSE139829* were downloaded from the GEO database (https://www.ncbi.nlm.nih.gov/geo). The data were filtered using R software and the Seurat package (version 4.1.1) to eliminate cells that do not meet the data quality standards. The number of highly variable genes was set at 3000. These 11 samples were integrated using "Harmony" (version 0.1.0). Uniform Manifold Approximation and Projection for Dimension Reduction (UMAP) method was used to reduce the dimension of data. Clustering of cells was conducted using the K-Nearest Neighbor (KNN) method with resolution=1.0. Cells were annotated using cell markers obtained from previous literature. Finally, the percentage of m^7^G-related genes in each cell was calculated by importing necroptosis genes using the “PercentageFeatureSet” function.

### Tumor immune infiltration analysis

In this study, we employed multiple methods for tumor immune infiltration. Including TIMER, QUANTISEQ, and so on [23, 24]. Immune checkpoint analysis was also performed to examine the immunological differences between the high-risk and the low-risk groups.

### Nomogram construction and ROC analysis

The nomogram of the risk score and relevant clinical information was depicted using the package Survival (version 3.2) and package rms (version 6.3).

### Cell culture, transfection, and qRT-PCR assay

MUM-2B, C918 cells were cultured in the RPMI1640 medium and OCM-1 cells in DMEM medium (Gibco, Shanghai, China) supplemented with 10% FBS (AuxGene, Gold Coast, Australia), transfected with siRNA (Ribobio, Nanjing, China) according to the manufacturer’s protocol. The sequences of the siRNAs we used are listed in Supplementary Table 1. Total cellular RNAs were extracted using Trizol™ Reagent (Life Technologies, CA, USA), and the reverse transcription and qRT-PCR assay were conducted using a reverse transcription kit and SYBR (Vazyme, Nanjing, China) according to the manufacturer’s protocol.

### Cell proliferation assay

5-ethynyl-2’-deoxyuridine (EdU) incorporation assay was performed to examine the cell proliferation capacity of the UVM cell lines. After incubation with EdU for 2 hours, cells were fixed with 4% Paraformaldehyde and stained with BeyoClick™ EdU Cell Proliferation Kit (Beyotime, Nanjing, China) according to the manufacturer’s protocol. A clustering Formation assay was also used to examine the proliferation ability of the cell lines. After transfection, the cells were seeded into a 6-well culture plate (Corning, NY, USA) and placed in a cell culture incubator for 10 days. The cells were then rinsed with PBS, fixed with methanol, stained with crystal violet, and eventually photographed using a digital camera (Canon).

### Scratch-wound healing assay

The scratch-wound assay is a simple, reproducible method commonly used to measure basic cell migration parameters. Cells were allowed to grow to confluence before a thin “wound” was introduced by scratching with a 10μL pipette tip. The cells subsequently began to migrate from the wound edge to fill the wound space. The wound size 24 hours after the introduction of the wound was recorded and quantified to assess the migration capacity of the cell lines.

### Transwell assay

Successfully transfected UVM cell lines were seeded into the upper well for 24 h and allowed to invade through the transwell plate (Corning, NY, USA). The cells on the inserts were fixed with methanol, stained with crystal violet, and counted under a light microscope to assess the migration capacity of the cells.

### Statistical analysis

*In vitro*, experimental data were processed using the SPSS software (version 26.0). All data were illustrated employing mean ± SD. One-way analysis of variance (ANOVA) and Turkey’s multiple comparisons of the means were employed in the analysis of multiple sets of data unless otherwise specified.

### Availability of data and materials

TCGA and GEO databases.

## RESULTS

**Figure 1:**
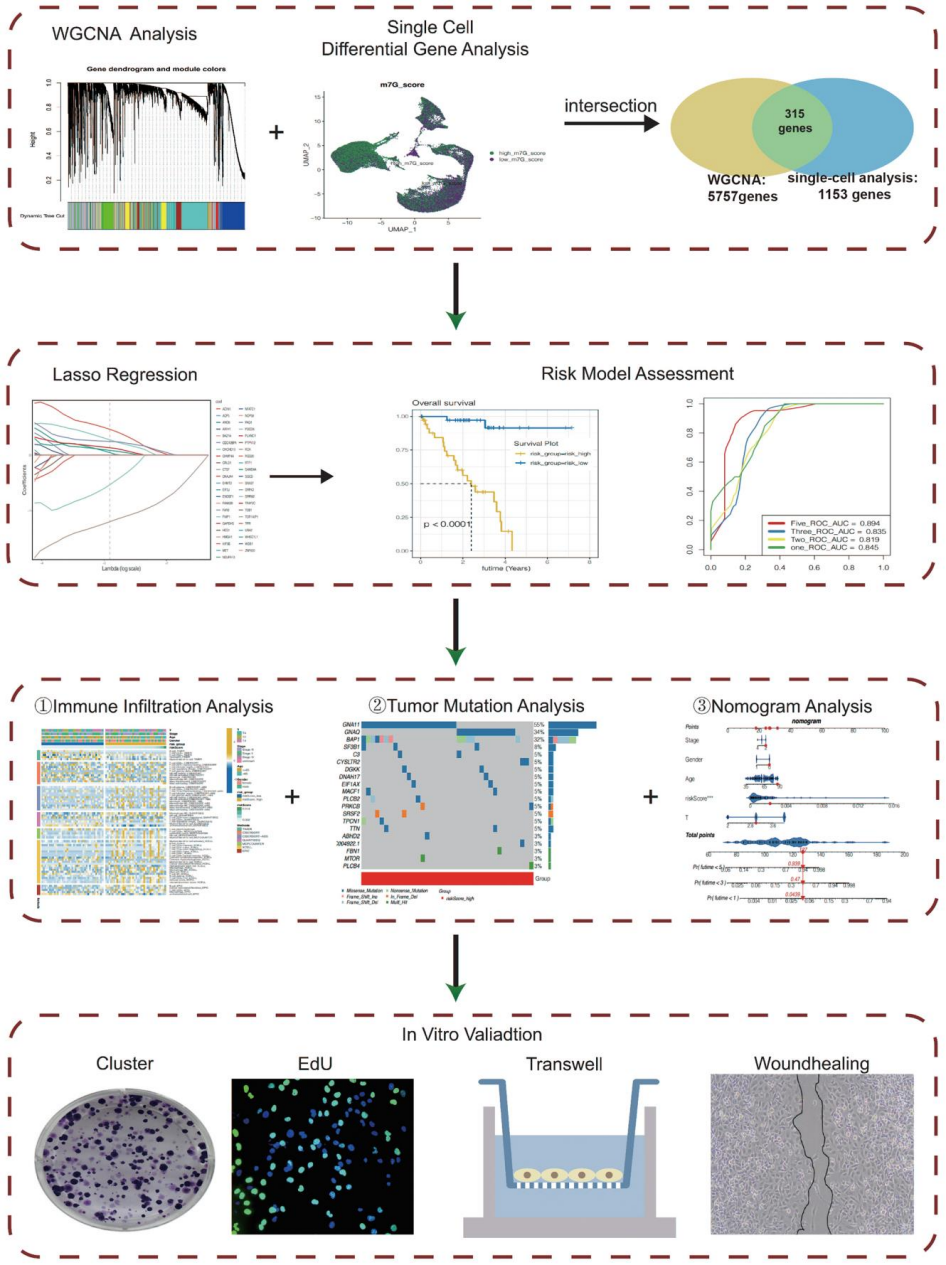
The flow chart.

Figure 1 shows our workflow.

### Single-cell analysis of the data

**Figure 2:**
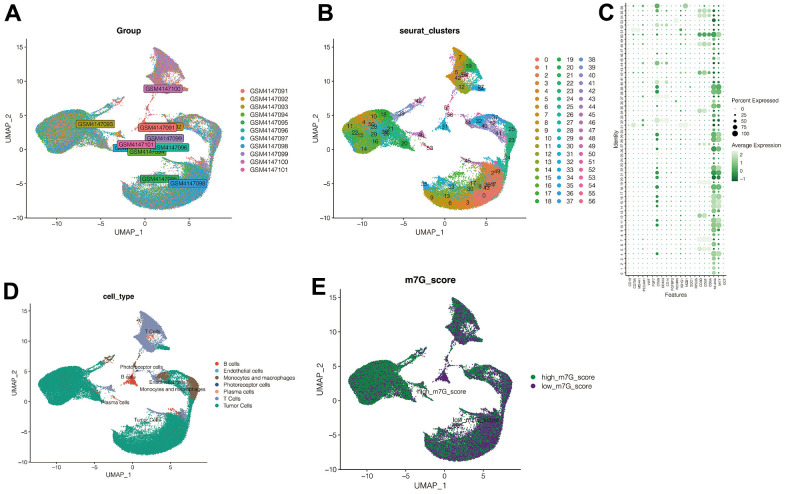
**Single cell sequencing analysis.** (**A**) No significant batch effects were found in the 11 samples in the single-cell dataset. (**B**) All cells were divided into 67 subclusters. (**C**, **D**) Cell annotation. (**E**) M7G scoring annotation. The cells were divided into high-M^7^G group and low-M^7^G group.

11 samples of UVM were included in our analysis. The batch effects between the 11 samples were negligible, and thus suitable for subsequent analysis (Figure 2A). After quality control and clustering analysis of the data, all cells were divided into 67 subclusters (Figure 2B). Based on the expression of the signature genes, all cells were annotated into five cell types: The B cells, Endothelial cells, Monocytes, macrophage cells, photoreceptor cells, Plasma cells, and T cells (Figure 2C, 2D), as well as the Tumor cells. As shown in Figure 2E, the activation of the m^7^G phenotype varied in different cell types. Using differential analysis, the genes with adjusted *p* -value<0.05 was set, and, | avg_log_2_FC |> 0.3 were selected and 1153 genes closely associated with the m7G phenotype were defined.

### WCGNA analysis filtered out modules and genes that are related to the m^7^G phenotype

**Figure 3:**
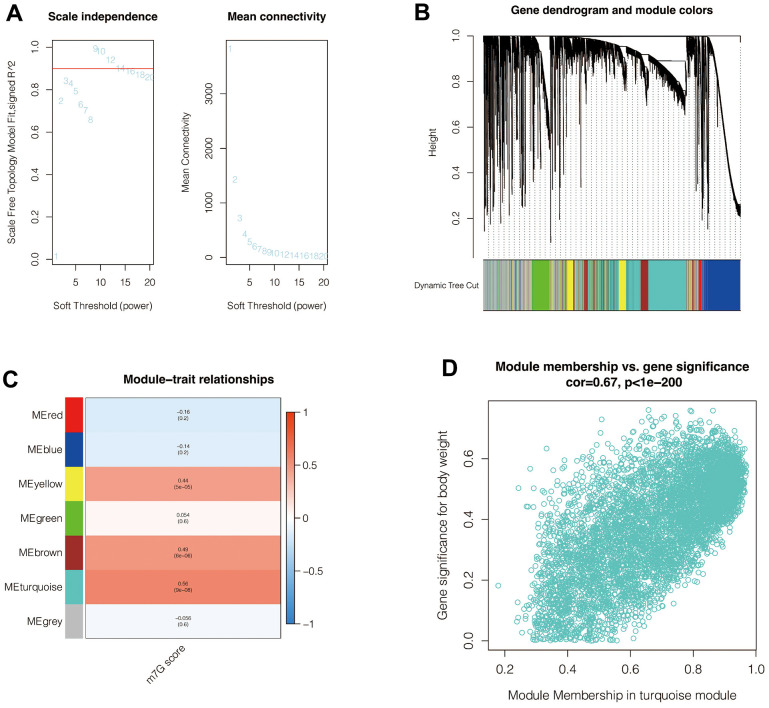
**Weighted co-expression network analysis.** (**A**) When setting a soft-zone threshold value reaches 9, R^2^ > 0.8, suggesting that the data fit a power-law distribution and were suitable for WGCNA analysis. Moreover, the mean connectivity fluctuates little as the soft domain value increases. (**B**, **C**) Clustering of modules. The turquoise module had the highest correlation with the M7G score. (**D**) The correlation coefficient between module membership correlation and gene significance for body weight was 0.67, with *p* <0.001.

The enrichment score (M7G score) of the M7G phenotype for each cell was first calculated by ssGSEA analysis. To further search for genes associated with the M7G phenotype, WGCNA analysis was performed. In Figure 3A, when setting a soft-zone threshold value reaches 9, R^2^ > 0.8, suggesting that the data fit a power-law distribution and were suitable for WGCNA analysis. Moreover, the mean connectivity fluctuates little as the soft domain value increases. As shown in Figure 3B, all the genes were collectively clustered into six non-gray modules, and it was found that among these modules (Figure 3C), the turquoise module had the highest correlation with the M7G score, with R = 0.56 and *p* <0. 001. In Figure 3D, the correlation coefficient between module membership correlation and gene significance for body weight was 0.67, with *p* <0.001. Therefore, the 5,757 genes from the turquoise module were selected and included in the subsequent analysis.

### The construction of a prognostic signature

**Figure 4:**
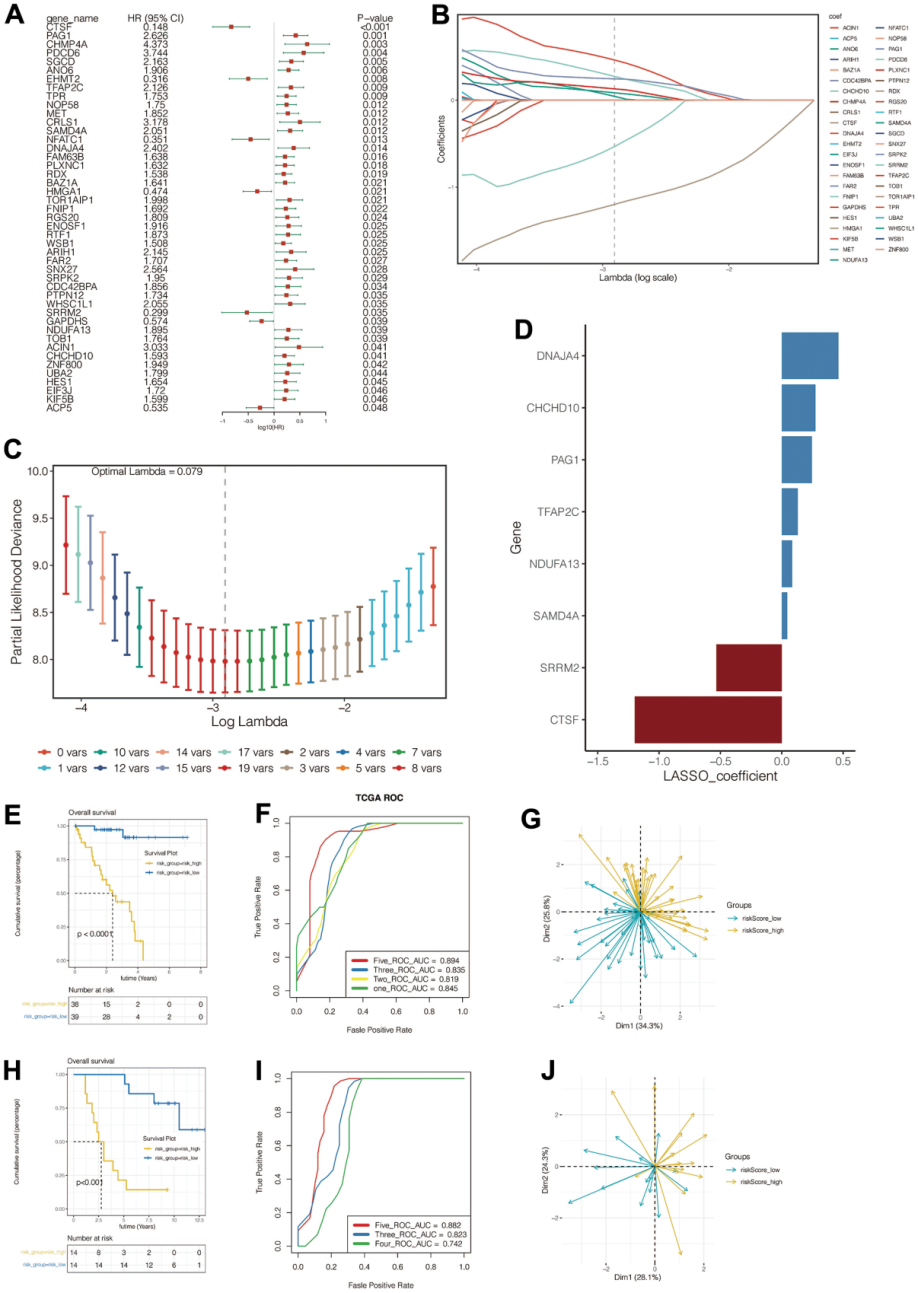
**Construction and evaluation of the prognostic signature.** (**A**) Univariate Cox identified genes associated with prognosis. (**B**–**D**) Lasso regression was used to construct the prognostic model. (**E**–**G**) Prognosis assessment, ROC curve construction and principal component analysis in training cohort. (**H**–**J**) Prognosis assessment, ROC curve construction and principal component analysis in validation cohort.

After intersecting the 1153 genes obtained from the single-cell data analysis with those obtained from the WGCNA analysis described above, a total of 355 genes were obtained. Eventually, 315 genes were identified to be suitable for further analysis. We then conducted a univariate cox analysis to filter the prognosis-related genes. We found that 45 genes were of significant prognostic value (*p*<0.05) (Figure 4A).

Subsequently, in the TCGA cohort, we further screened for genes associated with prognosis using the LASSO regression method. (Figure 4B, 4C) When the optimum Lambda is 0.055, A total of eight genes significantly associated with UVM prognosis were obtained, genes *CTSF*, *PAG1*, *TFAP2C*, *SAMD4A*, *DNAJA4*, *SRRM2*, *NDUFA13*, and *CHCHD10* were included in the prognostic signature. The signature was illustrated as follows:


$$\begin{array}{l} Risk\;Score=0.282*TPM_{DNAJA4}+0.196*TPM_{PAG1}+\\ 0.159*TPM_{CHCHD10}+0.048*TPM_{TFAP2C}+0.024*\\ TPM_{NDUFA13}+(-0.223)*TPM_{SRRM2}+(-1.058)*TPM_{CTSF} \end{array}$$

Figure 4D vividly depicted the genes and their corresponding coefficients. HR values of model genes were summarized in Supplementary Table 2.

All patients in the training cohort were divided into high risk-score and low risk-score groups according to the median value of the risk score. The high-risk group had significantly poorer outcomes (Figure 4E), and the model predicted that the area under the ROC curve (AUC) for patients’ 1,2,3,5-year mortality was 0.845, 0.819, 0.835, and 0.894, respectively (Figure 4F). Additionally, the model can precisely predict the outcomes of the patients with UVM in the validation cohort (downloaded from the GEO database *GSE84976*) (Figure 4H, 4I). Patients in the high-risk group had a poorer prognosis, and the model predicted the area under the ROC curve of 3,4,5 years was 0.823,0.742,0.882. PCA analysis further revealed the distribution of the patients in the PCA plot between the two groups varied both in the training cohort and the validation cohort. (Figure 4G, 4J).

### Immune infiltration and immune checkpoint analysis showed the risk score had strong immune correlations

**Figure 5:**
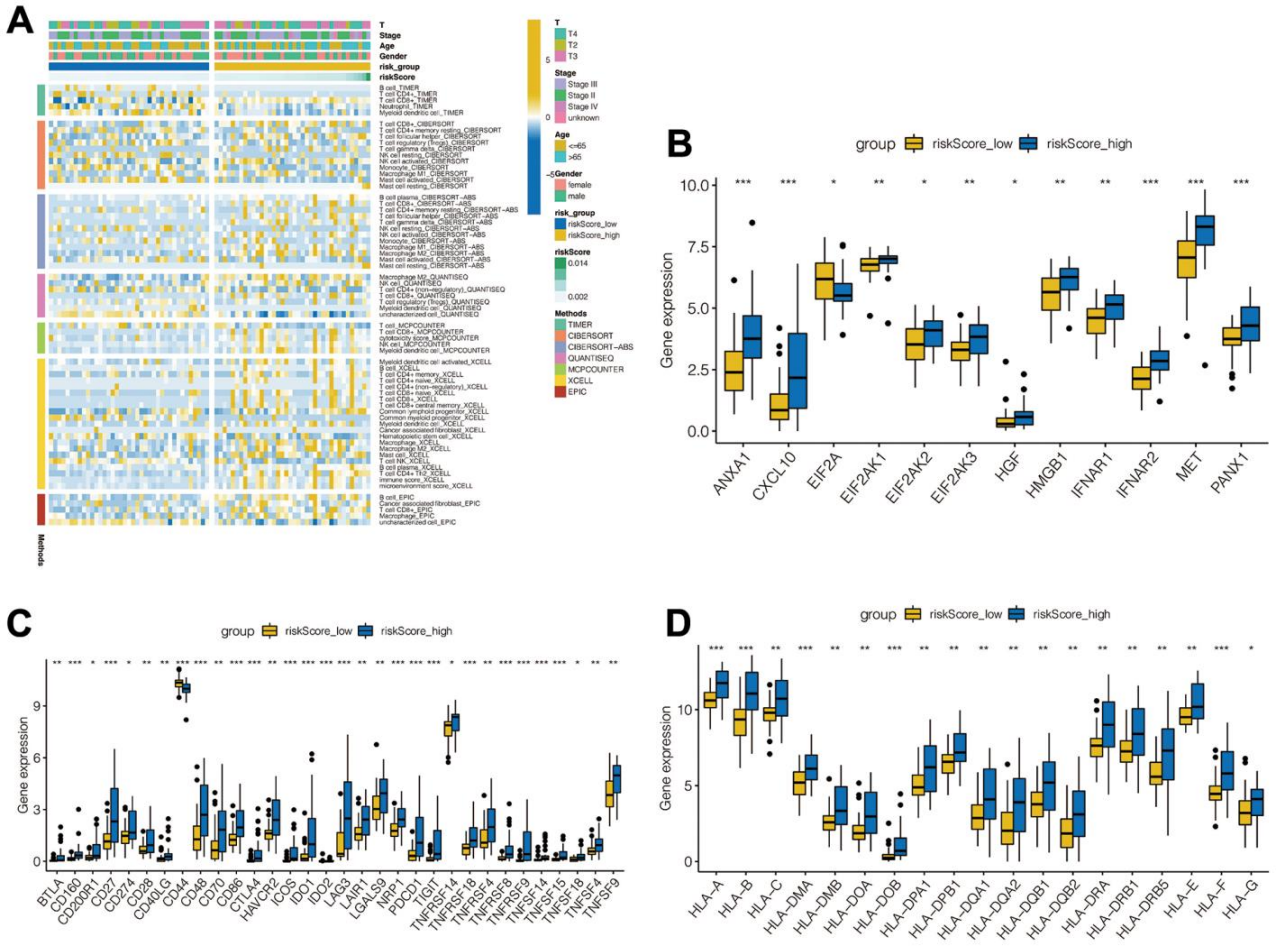
**Analysis of immune microenvironment.** (**A**) Immune landscapes between high-risk and low-risk groups. (**B**) Expression levels of immune checkpoint related genes between high-risk and low-risk groups. (**C**) Tumor necrosis factor-related gene expression levels between high - and low-risk groups. (**D**) Expression levels of leukocyte antigen related genes between high - and low-risk groups.

We further analyzed the tumor immune-microenvironment between the high-risk group and the low-risk group. Using multiple immune infiltration algorithms, we illustrated a case-based heatmap for the infiltration algorithms which scored significantly different between the two groups (Figure 5A). Furthermore, we compared the expression of 79 immune checkpoint genes acknowledged by previous researchers and performed Wilcoxon tests. (Figure 5B) [25]. As shown in Figure 5C, 5D, the expression of tumor necrosis factor and leukocyte antigen genes were all increased in the high-risk group when compared to the low-risk group.

### Tumor mutation analysis

**Figure 6:**
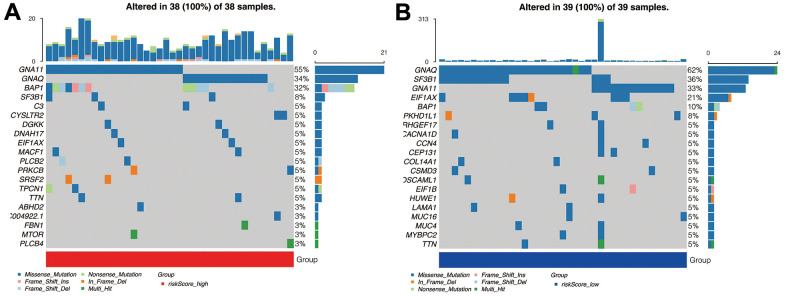
**Mutation analysis.** (**A**) The mutant landscape of the high-risk group. (**B**) The mutant landscape of the low-risk group.

In Figure 6A, in the high-risk group, the top five genes mutated were, respectively, *GNA11, GNAQ, BAP1, SF3B1,* and *C3*. In the low-risk group, however, the top five genes were, *GNA1, SF3B1, GNA11, EIF1AX,* and *BAP1* (Figure 6B). The commonly mutated genes are GNA11 and BAP1. Interestingly, gene GNAQ mutation, which is a classical alteration in the G_q_ pathways, is more commonly seen in the high-risk group, implying our signature’s underlying correlation with the G_q_ pathways.

### Construction of a nomogram

**Figure 7:**
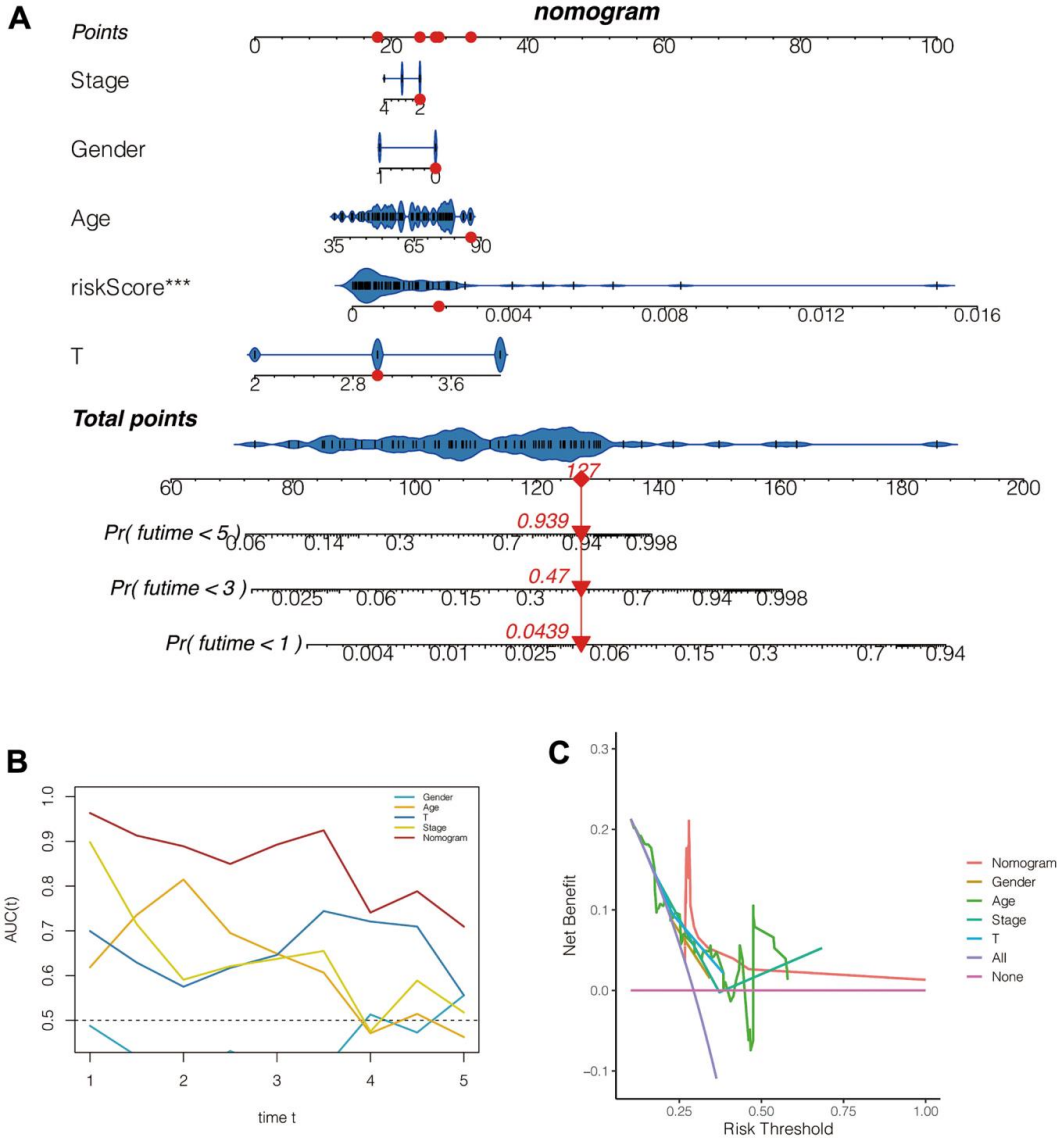
**Construction and evaluation of the nomogram.** (**A**) The nomogram. (**B**) The ROC curve. (**C**) The decision curve.

As shown in Figure 7A, the 1,3,5-year fatality of patient *TCGA-VD-AA8N* was 0.0439, 0.47, 0.939, respectively. Moreover, the ROC C-index was 0.849 *95%CI:(0.771, 0.928)*, suggesting the high accuracy of this nomogram in assessing patient prognosis. Besides, the continuous prognosis ROC analysis found that the area under the ROC curve of the Nomogram evaluation for patient prognosis was above 0.8, which was higher than the other clinical characteristics (Figure 7B). Moreover, as shown in Figure 7C, the clinical interventions of the patients according to the Nomogram benefit the patients better than the other clinical characteristics, such as gender, age, tumor size, and Stages.

### *In vivo* studies validated the oncogenic potential of gene *PAG1*

**Figure 8:**
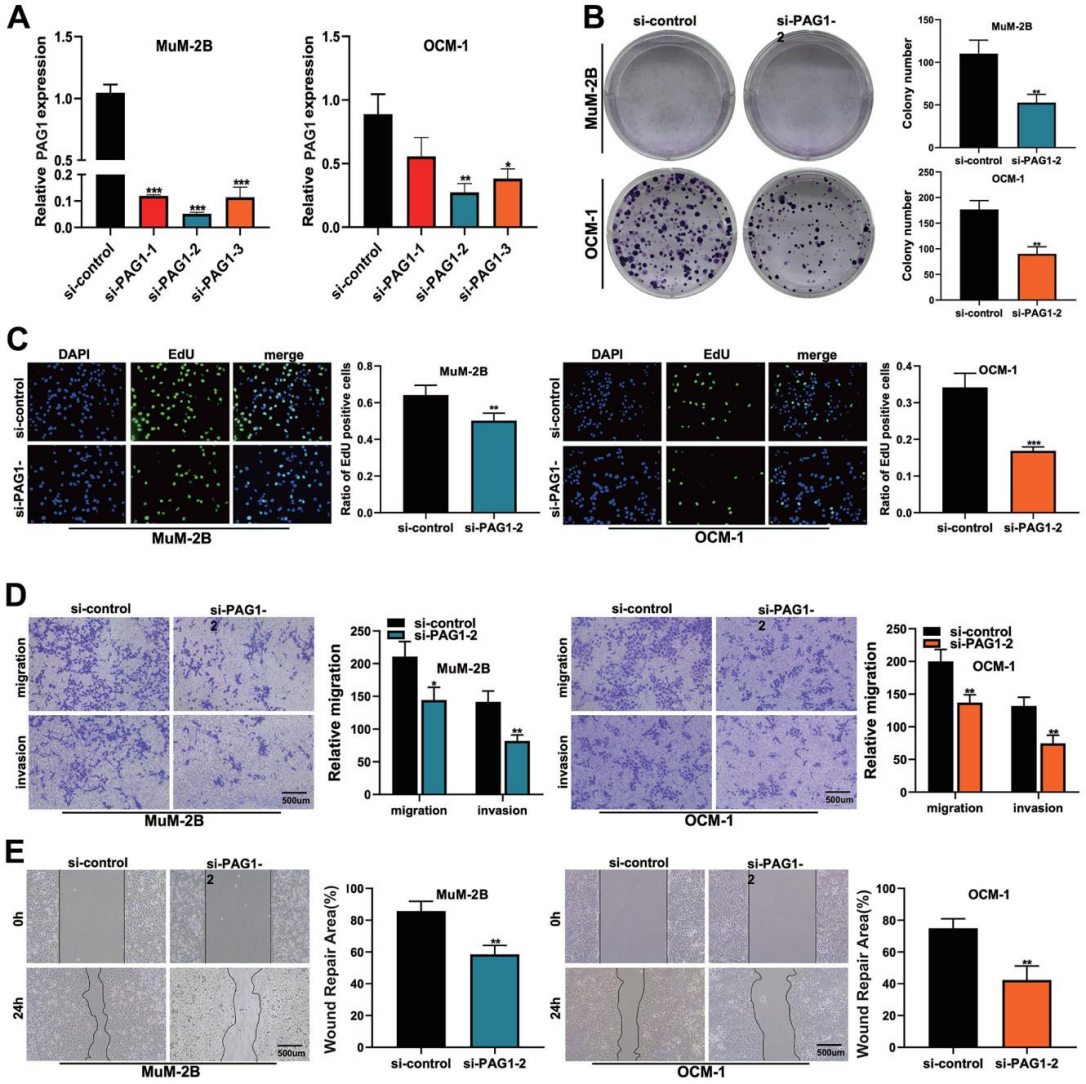
**The function of PAG1 in UVM cell line was verified by cell assay.** (**A**) Knockdown efficiency of PAG1 gene. The knockdown efficiency of si-PAG1-2 group was the highest. (**B**) Clone formation experiment. The proliferation ability of MUM-2B and OCM-1 cell lines decreased significantly after PAG-1 knockdown. (**C**) EdU assay. (**D**) Transwell experiment. The migration and invasion ability of MuM-2B cell lines and OCM-1 cell lines were significantly reduced after PAG-1 knockdown. (**E**) Wound healing experiment.

To further validate the robustness of our previously constructed risk model, we used *in vivo* experiments to validate the oncogenic role of a gene in the signature model in UVM patients, gene *PAG1*. Because the overexpression of *PAG1* could most significantly elevate the risk score of our previously constructed model, genetic manipulation on the mRNA level was performed. Firstly, we found that the expression of the *PAG1* gene was significantly suppressed after siRNA transfection in 3 variant cell lines (Figure 8A). Cell proliferation assays showed that after the knockdown of gene *PAG1*, MUM-2B, and OCM-1 cell lines showed a significantly lower rate of proliferation (Figure 8B, 8C). Scratch and wound healing assays showed that the migration capacity of MuM2B cells and OCM-1 cells were significantly compromised after the knockdown of gene *PAG1* (Figure 8D). Furthermore, the transwell assay showed that the number of cells which migrated through the transwell plate significantly reduced after *PAG1* knockdown (Figure 8E). Collectively, these results showed that the *PAG1* gene plays a crucial role in the proliferation and migration of the UVM cell lines.

## DISCUSSION

Uveal Melanoma derives from the uveal tract melanocytes, including the iris, ciliary body, and choroid [26]. Recent basic and clinical research has figured out, that Uveal melanoma was characterized by a series of genetic mutations like the G_q_ pathway alterations, the gene *BAP1*, splice, and *EIF1AX* mutations (also known as the BSE event) [27]. Despite the rapid advances in finding the crucial molecular events in UVM, many molecular mechanisms and underlying prognostic indicators are still left to be discovered. Moreover, although the diagnosis and treatment have progressed rapidly in recent years, the early diagnosis, early treatment, and the search for new prognostic markers are still lagging behind [28]. Therefore, new interventions and prognostic targets are urgently needed for scientists.

RNA methylation in cancer is being widely studied by researchers these days. The research of N7-Methylguanosine (m^7^G) in the fields of science is emerging and constantly making progress these days [29]. Orellana et al. found that METTL1, an important RNA methyltransferase, could catalyze N7-methylguanosine (m^7^G) modification of tRNAs in humans, inducing oncogenic transformation in cancer [30]. Therefore, targeting the m^7^G biological process could be a promising target in cancer therapy. However, little was known about the effect of m^7^ G-associated genes on Uveal melanoma.

In this study, we constructed an N7-Methylguanosine-associated prognostic gene signature on UVM and identified a gene with the highest hazard ratio (HR), *PAG1*, which might be a potential regulator of UVM progression. Due to the limited number of genes that are correlated with the biological process of m^7^G, an alternative method was used to construct a gene signature. Using WGCNA, we identified the module most correlated with m7G, and the signature model was constructed accordingly.

*PAG1* is a member of the Src family [31, 32]. *PAG1* is a ubiquitously expressed transmembrane adaptor protein, which helps recruit cytoplasmic C-terminal Src kinase (CSK) to lipid raft-associated Src kinases, thus interacting with several other important cytoplasmic and plasma membrane-associated proteins [33].

*PAG1* has been proved to be essential for the development of various kinds of malignancies, including Laryngeal carcinoma and nasopharyngeal carcinoma. However, little is known about the effect of *PAG1* on UVM. By identifying an m^7^G-associated signature, we selected *PAG1* for further analysis to test whether the gene could take part in the progression of UVM.

In general, our study searched for a related gene module by the WGCNA method and established a suitable prognostic model, which was validated with *PAG1* gene knockdown *in vitro* experiments. Because no previous literature claims that m7G-related genes can construct a suitable prognostic model in UVM, this study demonstrates that the model constructed by genes related to the biological process of m7G is related to the survival of UVM patients. This further suggests that m7G may play an important role in the occurrence and development of UVM.

## CONCLUSIONS

Our study is the first to construct an m7G-related prognostic model in Uveal melanoma. With this prognostic model, the prognosis and immune status of UVM patients can be well evaluated. Furthermore, we identified a novel biomarker: *PAG1*.

## Supplementary Material

Supplementary Tables
