# Supplementary material for: Single cell sequencing analysis constructed the N7-methylguanosine (m7G)-related prognostic signature in uveal melanoma
Source: Aging (Albany NY). 2023 Mar 14;15(6):2082–96. doi: 10.18632/aging.204592 (PMC10085590; doi:10.18632/aging.204592)
Supplement: Supplementary Tables [file aging-15-204592-s001.pdf]

SUPPLEMENTARY TABLES

Supplementary Table 1. The siRNA sequence of PAG1.

H55824-siPAGI-1-SS	CAUGCAGCAUUACGAGGAATT
H55824-siPAG1-1-as	UUCCUCGUAAUGCUGCAUGTT
H55824-siPAG1-2-ss	GCCUGAUUAUGAAGCGAUATT
H55824-siPAG1-2-as	UAUCGCUUCAUAAUCAGGCTT
H55824-siPAG1-3-ss	GCUAUGUACUCAUCAGUAATT
H55824-siPAG1-3-as	UUACUGAUGAGUACAUAGCTT

Supplementary Table 2. The HR and p values of the model genes.

Gene symbol	HR	P value
DNAJA4	2.402	0.014
CHCHD10	1.593	0.041
PAG1	2.626	0.001
TFAP2C	2.126	0.009
NDUFA13	1.895	0.039
SAMD4A	2.051	0.012
SRRM2	0.299	0.035
CTSF	0.148	<0.001
